# Special Issue: Cyclodextrins: Properties and Applications, 2nd Edition

**DOI:** 10.3390/ijms262411789

**Published:** 2025-12-05

**Authors:** Miguel A. Esteso, Carmen M. Romero

**Affiliations:** 1 Facultad de Ciencias de la Salud, Universidad Católica de Ávila, Calle los Canteros s/n, 05005 Ávila, Spain; 2 U.D. Química Física, Universidad de Alcalá, 28805 Alcalá de Henares, Spain; 3 Departamento de Química, Facultad de Ciencias, Universidad Nacional de Colombia, Sede Bogotá, Carrera 30 No. 45-03, Bogotá 111311, Colombia

## 1. Introduction

Cyclodextrins (CDs) are cyclic oligosaccharides with a supramolecular structure obtained from the enzymatic degradation of starch. The three natural cyclodextrins, α-, β-, and γ-CD, containing six, seven, and eight glucose units, respectively, are the most common and extensively studied. Cyclodextrins are crystalline, non-hygroscopic, homogeneous substances that are soluble in water due to their hydrophilic outer surface with hydroxyl groups at the ends, and a central hydrophobic cavity of variable size in a truncated cone shape, into which nonpolar or hydrophobic molecules can be incorporated [1,2,3,4,5]. The dual hydrophilic and hydrophobic character of these compounds allows them to form inclusion complexes with a wide variety of hydrophobic guest molecules, involving ‘host–guest’-type intramolecular interactions.

The main interest in CDs lies in their ability to form inclusion complexes with several solid, liquid, and gaseous compounds. Guest molecules form non-covalent complexes with CDs through various types of interactions, including van der Waals forces, hydrophobic interactions, and hydrogen bonds, without the formation or breaking of covalent bonds. These interactions can lead to the formation of inclusion complexes, partial inclusion complexes, and CD couplings, as well as CDs containing supramolecular hydrogels and supramolecular micelles. The properties of the resulting products mean that they can be used widely in food, medicine, cosmetics, and environmental protection, among other areas, as has been shown by the increasing number of industrial applications since the 1980s [1,4,5,6,7,8,9,10,11,12].

Cyclodextrins are versatile supramolecular hosts with unique inclusion capabilities. Their non-symmetrical toroidal (truncated cone) structure enables them to encapsulate and protect guest molecules, meaning that they have a wide range of applications across pharmaceuticals, food, cosmetics, new materials, and environmental industries.

Cyclodextrins’ encapsulation capacity and flexibility allow host−guest interactions to modify the physical, chemical, and biological properties of the guest molecules, improving several of the resulting substance’s properties, such as its bioavailability, the physical and chemical stability of the material, the shelf life of the product, and the control of the release of important substances in foods or medicines. Their solubility in water is fundamental in the discovery of new developments and applications of CDs. The water solubility of CDs can easily be increased by the formation of CD inclusion complexes, whose solubility is usually greater than that of the nonpolar or hydrophobic molecules that form the complex alone [7].

A deep understanding of the structural and physicochemical properties of CDs, and in particular, an awareness of how non-covalent interactions work and how strong they are, is essential to optimize the development of the various applications of these supramolecular compounds, and to carry out processes with high efficiency at molecular level. Several current studies refer to the modification of CDs using a variety of methods. The inclusion complexes formed are frequently produced by the amination, esterification, or etherification of primary and secondary hydroxyl groups of the CDs.

Cyclodextrins are the object of numerous studies, both fundamental and applied. They continue to be of interest to many researchers for both their fundamental and technological applications in several industries, and several current studies refer to the modification of CDs and their physicochemical behavior. They also discuss different applications of CDs, such as in the medical, pharmaceutical, cosmetics [1,12,13,14,15], food [1,16,17,18], and textile industries [1,19], and in different processes related to biotechnology, agriculture, and the environment. CDs are also frequently used in controlled release systems to improve the solubility and bioavailability of poorly water-soluble substances, to control the chemical activity of guest molecules, and as catalysts for different reactions [20,21]. Recently, they have attracted interest in new fields such as the synthesis of innovative biomedical materials, and in the application of the properties of certain CD derivatives in interactions with the membranes of specific cells, increasing the potential of gene therapy [1].

The ability of a CD to form inclusion complexes with guest molecules depends on the chemical and physical characteristics of the CD, the size of the guest molecule, and the physicochemical interactions between the CD, the guest, and the solvent, which is usually water. The chemical and physical characteristics of the CD derivatives are affected by the position of the attached substituent inside the CD molecule, its structure, and the quantity of substituent groups per CD molecule. It should be pointed out that these interactions are highly influenced by solvation, temperature, and the selected solvent [22,23].

The previous Special Issue, Cyclodextrins: Properties and Applications [24], published in 2024, presents research papers and comprehensive reviews that focus on the advances in the knowledge of the structure, properties, and theoretical and practical applications of CDs. This Special Issue, Cyclodextrins: Properties and Applications, 2nd edition, continues with this goal, compiling nine papers that present new contributions regarding the possibilities of developing new CD-based products and their applications.

## 2. An Overview of Published Articles

This Special Issue, Cyclodextrins: Properties and Applications 2nd Edition, includes articles that refer to topics related to aspects of the theoretical and practical applications of CDs, focusing on design, synthesis, characterization, and especially on new developments and applications of these versatile compounds—see the list of contributions.

Contribution 1 achieves significant results regarding the complexation ability of CDs. It proposes a novel boron detection method based on current amplification through 1:1 binding between boron and a Fc/catechol-functionalized β-CyD inclusion complex. This method can detect boron in the presence of various anions or cations and has the potential to be applicable for monitoring boron concentrations in drinking water and wastewater.

Contribution 2 presents a study of the host–guest interaction and inclusion complexation of cinnamic acid with randomly methylated β-cyclodextrin. The inclusion complex obtained is highly soluble in water, thereby overcoming the poor bioavailability of cinnamic acid, and is characterized in solution and in a solid state. Experimental and theoretical results show that the complex formed is stable, supporting its use for clinical applications.

Contribution 3 shows how cyclodextrins (CDs) are host molecules frequently used to form inclusion complexes with different guests, such as N_2_, CO_2_, NH_3_, and N_2_O, among other small molecules. The results confirm the capacity of α-CD to store N_2_O, the thermal stability of the complex formed, and the applications of these types of complexes in the food and beverage industries.

Contribution 4 develops a new oral matrix tablet formulation, based on Kollidon^®^SR and chitosan, to optimize the low-dose oral bioavailability of chlorzoxazone, a non-steroidal anti-inflammatory drug. The results show that the three formulations present optimum pharmaco-technical properties and in vitro kinetic behavior, and have great potential to be used for the controlled delivery of chlorzoxazone.

Contribution 5 analyzes how to maximize the biological effects of vitamin D3 by complexing it within cyclodextrin nanosponges. The results show that complexation improves the biological function and bioavailability of vitamin D3 in the intestine and suggests that oral administration in humans can be considered a viable therapeutic strategy to improve the effects of treatment under conditions of low vitamin levels.

Contribution 6 is focused on the application of β and γ-cyclodextrins to form inclusion complexes with anticancer selenium-containing compounds consisting of different nonsteroidal anti-inflammatory drug (NSAID) derivatives with Se. The results show the increased solubility of two of the complexes formed. Theoretical models were used, and docking studies predicted the most stable host–guest interaction for the two best-rated complexes in this study.

Contribution 7 is a review that highlights the most relevant characteristics of cyclodextrins and their derivatives, and summarizes their applications, with an emphasis on the development of novel therapeutic approaches as drug delivery systems in neurodegenerative disorders. The manuscript suggests that future research should focus on refining CD formulations to ensure effective penetration of the BBB, enhance the stability of neuroactive drugs, and achieve targeted delivery to specific brain regions.

Contribution 8 is a review that enhances the importance of using cyclodextrins in the separation of enantiomers in food, pharmaceuticals, and pesticides, among other industries. The paper includes the recent theoretical background behind enantioseparation, factors that affect the efficacy of this process, and advancements in the application of β-cyclodextrin as a chiral selector. The authors conclude that the enantiomers of the selected compounds could be effectively separated using β-cyclodextrin under specific conditions that involve the physicochemical properties of the solute, the type of interaction involved in the formation of stable complexes with β-CD, and the experimental conditions of analysis.

Contribution 9 reviews the use of cyclodextrins as potential drug carriers of selenium and tellurium due to their ability to enhance their bioactivities and therapeutic properties. Selenium and tellurium compounds were selected due to their importance in the design and development of novel bioactive compounds to treat several pathologies. The review shows that cyclodextrins improve their poor solubility, stability, and toxicity and enhance their efficacy as antitumor and antiparasitic treatments.
